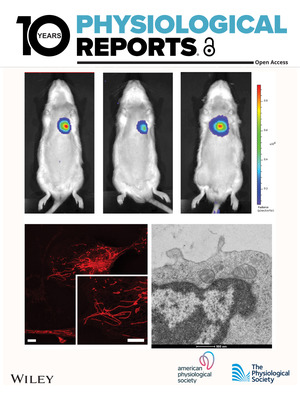# Cover Image

**DOI:** 10.14814/phy2.16049

**Published:** 2024-05-21

**Authors:** Bridget Nieto, Michael W. Cypress, Bong Sook Jhun, Jin O‐Uchi

## Abstract

The cover image is based on the Short Report *Adeno‐associated virus‐based approach for genetic modification of cardiac fibroblasts in adult rat hearts* by Bridget Nieto et al., https://doi.org/10.14814/phy2.15989